# Interactions between host biogenic amines and sand fly salivary yellow-related proteins

**DOI:** 10.1186/s13071-020-04105-2

**Published:** 2020-05-07

**Authors:** Tatiana Spitzova, Petra Sumova, Vera Volfova, Nikola Polanska, Luisa Poctova, Petr Volf

**Affiliations:** Department of Parasitology, Faculty of Science, Charles University, Vinicna 7, 128 44 Prague 2, Czech Republic

**Keywords:** *Phlebotomus argentipes*, *Sergentomyia schwetzi*, Serotonin, Histamine, Oviposition, Mortality, Yellow-related proteins, Anti-saliva antibodies

## Abstract

**Background:**

During blood feeding, sand flies inoculate salivary proteins that interact with the host haemostatic system. The blocking of biogenic amines such as serotonin and histamine helps to limit vasodilatation and clot formation, and thus enables the insect to finish the blood-feeding process. In sand flies, an amine-binding ability is known only for the yellow-related proteins of *Phlebotomus* and *Lutzomyia* vectors, but not yet for members of the genus *Sergentomyia*.

**Methods:**

The ability of *Phlebotomus argentipes* and *Sergentomyia schwetzi* recombinant yellow-related salivary proteins to bind histamine and serotonin was measured by microscale thermophoresis. Both sand fly species were also fed through a chicken-skin membrane on blood mixed with histamine or serotonin in order to check the effects of biogenic amines on sand fly fitness. Additionally, fecundity and mortality were compared in two groups of *P. argentipes* females fed on repeatedly-bitten and naive hamsters, respectively.

**Results:**

The *P. argentipes* recombinant yellow-related protein PagSP04 showed high binding affinity to serotonin and low affinity to histamine. No binding activity was detected for two yellow-related proteins of *S. schwetzi*. Elevated concentrations of serotonin significantly reduced the amount of eggs laid by *P. argentipes* when compared to the control. The fecundity of *S. schwetzi* and the mortality of both sand fly species were not impaired after the experimental membrane feeding. Additionally, there were no differences in oviposition or mortality between *P. argentipes* females fed on immunized or naive hamsters.

**Conclusions:**

Our results suggest that in natural conditions sand flies are able to cope with biogenic amines or anti-saliva antibodies without any influence on their fitness. The serotonin binding by salivary yellow-related proteins may play an important role in *Phlebotomus* species feeding on mammalian hosts, but not in *S. schwetzi*, which is adapted to reptiles.
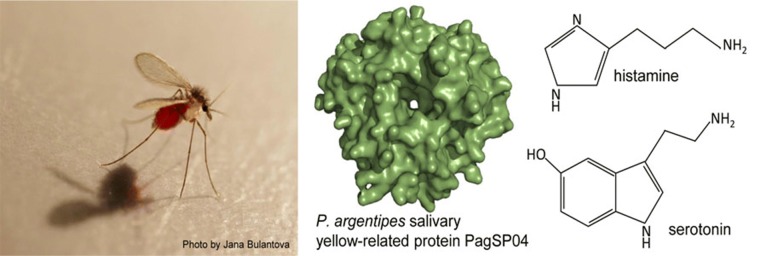

## Background

Females of phlebotomine sand flies (Diptera: Psychodidae) feed on blood in order to complete egg development. During blood-feeding they inoculate salivary proteins into the skin that counteract the host haemostatic system [1]. Biogenic amines, such as, histamine and serotonin, are crucial molecules for host haemostasis. Histamine is commonly associated with an immediate-type hypersensitivity response (i.e. increased vascular permeability and vasodilatation) and chemoattractant activity. This amine is produced by a wide variety of cell types (e.g. mast cells, basophils) [2]. Serotonin plays a role in numerous physiological processes, among others as an inflammatory modulator, vasoconstrictor and contributor to clot formation. Circulating platelets are the main storage site for peripheral serotonin [3].

Bloodsucking arthropods have developed various salivary molecules to cope with biogenic amines such as lipocalins in ticks [4] and triatomids [5] and D7 proteins in mosquitoes [5]. In sand flies, the ability to bind host biogenic amines has only been described for the family of salivary yellow-related proteins (YRPs) [6, 7]. Proteins of this family are found exclusively in insects and are abundant in phlebotomine sialomes with high variability in the number of YRPs among different sand fly species [1, 8]; they show a similar structure with some intraspecific modifications that influence the ligand-binding abilities [9].

In repeatedly-bitten hosts, sand fly saliva also stimulates the production of high levels of species-specific anti-saliva antibodies [1]. According to some authors, these antibodies could have an impact on sand fly fecundity and mortality [10–12]; however, other studies have not found any significant effects [13–15].

In this study, we focused on two sand fly species, *Phlebotomus argentipes* and *Sergentomyia schwetzi*. *Phlebotomus* (*Euphlebotomus*) *argentipes* is the most important vector of visceral leishmaniasis in Asia [16], with a mainly zoophilic feeding behaviour and a preference to feed on humans as the second choice [17]. This study of the amine-binding properties of its yellow-related protein adds to previously published data on other visceral leishmaniasis vectors in America, Europe and Africa [6, 7]. *Sergentomyia schwetzi* is the only representative of the genus *Sergentomyia* available in laboratory colonies worldwide [18]. *Sergentomyia* species prefer to feed on reptiles [19], and to our knowledge, this is the first study to describe the *S. schwetzi* salivary yellow-related proteins and their role in feeding processes.

The main aims of the study were (i) to compare the ability of *P. argentipes* and *S. schwetzi* yellow-related proteins to bind biogenic amines, particularly histamine and serotonin; (ii) to clarify if the fecundity and mortality of *P. argentipes* and *S. schwetzi* could be affected by biogenic amines present in blood using membrane feeding; and (iii) to study if high levels of anti-*P. argentipes* saliva antibodies in repeatedly-bitten hamsters interfere with *P. argentipes* fecundity and mortality.

## Methods

### Sand flies and laboratory rodents

Laboratory colonies of *P. argentipes* originating from India and *S. schwetzi* originating from Ethiopia were maintained in the insectary of the Department of Parasitology, Charles University, under standard conditions (at 26 °C, fed on 50% sucrose, with a 14 h:10 h light:dark photoperiod) as described by Volf and Volfova [20]. The hamsters used were 3-month-old Syrian hamsters (*Mesocricetus auratus*) kept in the animal facility of the Department of Parasitology, Charles University.

### Expression of recombinant yellow-related proteins

For biogenic amine-binding experiments, one *P. argentipes* and two *S. schwetzi* salivary yellow-related proteins were expressed in a human cell line (Table 1). The gene constructs were prepared by isolating the total RNA from one-day-old females using a High Pure RNA Tissue Kit (Roche, Prague, Czech Republic), then the cDNA was synthesised with the anchored-oligo (dT)_18_ primers using the Transcriptor First Strand cDNA Synthesis Kit (Roche) following the manufacturer’s protocol. The requested transcripts were amplified from cDNA by PCR and subcloned into the pTW5sec expression plasmid, a derivative of pTT5 [21, 22]. Proteins expressed using this plasmid contain additional ITG- and -GTHHHHHHHHG amino sequences at their N- and C-termini, respectively. Proteins were then transiently expressed in the human embryonic kidney 293S (HEK293S) GnTI- cell line (ATCC CRL-3022), as previously described [6, 21, 23].

**Table 1:** Recombinant salivary yellow-related proteins

Name	Species	MW (kDa)	ε (M^−1^cm^−1^)	GenBank ID
PagSP04	*P. argentipes*	44.92	62020	ABA12136.1
SschwYRP1	*S. schwetzi*	42.51	57090	QHO60691.1
SschwYRP3	*S. schwetzi*	45.34	70103	QHO60693.1

*Notes*: List of recombinant yellow-related proteins based on the salivary proteins of *P. argentipes* and *S. schwetzi*. The name, species, molecular weight (MW), extinction coefficient (ε) and GenBank accession numbers are indicated

All recombinant yellow-related proteins were purified by IMAC chromatography using HiTrap Talon Crude columns (GE Healthcare, Prague, Czech Republic) followed by size exclusion chromatography using Superdex 200 Increase 10/300 GL column (GE Healthcare). Proteins were subsequently stored in phosphate-buffered saline (PBS; pH 7.5). Protein concentrations were measured using a NanoDrop ND-1000 spectrophotometer (Thermo Fisher Scientific, Prague, Czech Republic) at 280 nm and calculated using the theoretical molar extinction coefficients and molecular weights of the proteins (Table 1). The identity and purity of the proteins were further verified by mass spectrometry.

### Microscale thermophoresis

Microscale thermophoresis (MST) was used to measure the binding affinities between recombinant yellow-related proteins and their potential ligands, serotonin and histamine. The MST affinity experiments were performed as described in [6] with minor modifications.

The highly pure recombinant yellow-related proteins were fluorescently labelled by a Monolith His-Tag Labeling Kit RED-tris-NTA 2nd Generation (Nanotemper, Munich, Germany) according to the manufacturer’s instructions. Fluorescent YRPs were then diluted to 40 nM concentration (corresponding to 1.73 µg/ml) in the MST buffer (50 mM Tris-HCl, pH 7.4; 150 mM NaCl; 10 mM MgCl_2_; 0.05% Tween-20) and centrifuged for 10 min at 15,000×*g* at 4 °C to remove protein aggregates. Serotonin (Sigma-Aldrich, Prague, Czech Republic) and histamine (Sigma-Aldrich) were dissolved in MST buffer. For each tested recombinant YRP, a titration series with a constant concentration of fluorescently labelled YRP and an equal amount of a two-fold dilution series of a single unlabelled ligand were prepared in the MST buffer. Binding experiments were performed on a Monolith NT.115 PicoRed (Nanotemper).

### Membrane feeding with histamine and serotonin

*Phlebotomus argentipes* and *S. schwetzi* females (5–7 days-old) were fed through a chick-skin membrane by the standard method described by Volf & Volfova [20]. From 100 to 120 female sand flies were used for each group. Histamine and serotonin were dissolved in 200 μl of physiological saline to concentrations of 0.3 mg/ml and 0.07 mg/ml, respectively, and mixed with 3 ml of defibrinated rabbit blood. In order to emphasize the effect of biogenic amines on the sand fly fitness, we decided to use elevated “non-physiological” concentrations for both amines [24, 25]. A blood mixture with saline only was used as a negative control. The experiment with serotonin and *P. argentipes* was repeated twice, to confirm differences between the experimental and naive groups. Engorged sand flies were maintained in cages under standard conditions until defecation.

### Feeding on repeatedly exposed hamsters

Hamsters of both sexes were randomly assigned to two groups of 6 animals each. In the first group, anesthetized animals (ketamine 50 mg/kg and xylazine 2 mg/kg, intramuscularly) were exposed to 100–290 *P. argentipes* females six-times at 7–15-day intervals. The second group served as a negative control. One week after the last exposure, hamsters from each group were exposed to 100 *P. argentipes* females (5–7 days-old) for 45 min. Engorged sand flies were maintained in cages under standard conditions until defecation.

Sera were collected from anesthetized animals from both groups one week after the last exposure to sand flies and stored at − 80 °C until use.

### Oviposition and mortality monitoring

After defecation (3 and 5 days after blood-feeding for *P. argentipes* and *S. schwetzi*, respectively), females were individually separated into small glass vials equipped with wet filter paper, closed with fine gauze and allowed to oviposit. All vials were placed into a single plastic box with its base filled with wet filter paper to ensure a uniform microclimate [18]. The humidity, mortality and occurrence of eggs were checked daily for the next 5 days, and laid eggs were counted at the end of the experiment.

### Detection of anti-*P. argentipes* IgG

Anti-*P. argentipes* IgG were measured by an enzyme-linked immunosorbent assay (ELISA) as described in [26] with minor modifications. Briefly, microtiter plates were coated with salivary gland homogenate (SGH) (0.2 salivary gland per well) obtained as described in [26]. Hamster sera were diluted 1:100 in 2% (w/v) low fat dry milk with 0.05%Tween-20 (PBS-Tw), and secondary antibodies (anti-hamster IgG, AbD Serotec) were diluted 1:1000 in PBS-Tw. Each serum was tested in duplicate. Absorbance values were reported as optical densities (ODs) with a subtracted blank (value in the control wells).

### Statistical analysis

Statistical analyses were carried out using R software (http://cran.r-project.org/). Differences in oviposition between groups were tested by fitting generalised linear models (GLM) with quasi-poisson distribution. Differences in mortality between groups were analysed by a 2-sample test for equality of proportions. A *P*-value of < 0.05 was considered to indicate statistical significance.

For the MST experiments, the Kd (dissociation constant) model binding curves were fitted to the average of three independent repetitions of each measurement. The Kd-values, confidence intervals, amplitudes and the signal-to-noise levels were calculated using the NanoTemper analytical software package.

## Results

### Ligand binding analysis using microscale thermophoresis

**Fig. 1:**
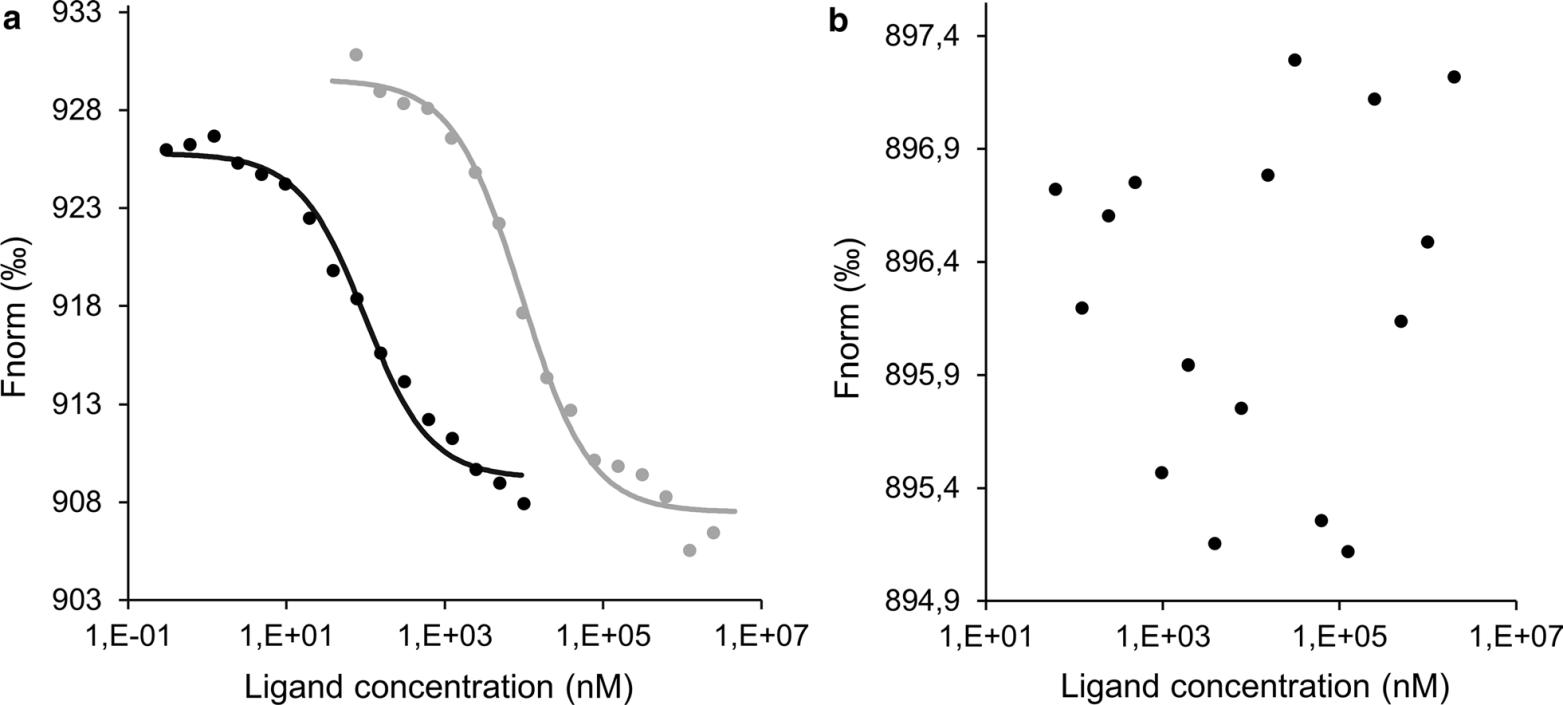
Amine-binding properties of *Phlebotmus argentipes* and *Sergentomyia schwetzi* yellow-related proteins. **a** Kd model binding curves of *P. argentipes* yellow-related protein. The binding curves for serotonin and histamine are depicted in black and grey, respectively. Each curve and data point represent the average of three independent experiments. **b** An example of non-measurable binding interaction for *S. schwetzi* yellow-related proteins

The amine-binding properties of *P. argentipes* and *S. schwetzi* YRPs measured by MST are visualized in Fig. 1, and the binding parameters are summarized in Table 2. *Phlebotomus argentipes* yellow-related protein PagSP04 bound serotonin with high affinity (Kd = 86.9 nM), while it had only a low affinity for histamine (Kd = 9.3 µM). On the contrary, neither of the *S. schwetzi* YRPs tested had detectable binding affinities for either biogenic amine.

**Table 2:** Amine-binding properties of recombinant yellow-related proteins

YRP	Ligand	Kd (nM)	Kd 95% CI	Amplitude	Signal to noise
PagSP04	Serotonin	86.9	72.5–101.3	16.6	20.8
	Histamine	9304.5	8042.9–10566.1	22.1	23.7
SschwYRP1	Serotonin	nb	na	na	na
	Histamine	nb	na	na	na
SschwYRP3	Serotonin	nb	na	na	na
	Histamine	nb	na	na	na

*Notes*: Dissociation constants (Kd; nM), Kd 95% confidence intervals (95% CI), amplitudes and the signal-to-noise ratios for *P. argentipes* and *S. schwetzi* yellow-related proteins

*Abbreviations*: nb, non-measurable binding interaction, na, not applicable

### Feeding on biogenic amines: effects on sand fly oviposition and mortality

Results from the experimental feeding of *P. argentipes* and *S. schwetzi* on histamine and serotonin are summarized in Table 3. In total, 358 *P. argentipes* and 178 *S. schwetzi* females were separated individually into glass vials to monitor oviposition and mortality. The prevalence of ovipositing *P. argentipes* and *S. schwetzi* females reached very similar numbers regardless of whether sand flies were fed on blood mixed with amines or blood mixed with physiological solution, with percentages ranging between 85–96%. However, the *P. argentipes* females fed on blood mixed with serotonin showed a significant decrease in the number of laid eggs compared to the control group (*t* = − 4.46, *df* = 195, *P* < 0.001). The median numbers of eggs laid by the serotonin and control groups were 32 (range: 1–62) and 43 (range: 1–74), respectively. In the other experimental groups, the number of laid eggs was not significantly affected by the presence of biogenic amines in the blood meal.

**Table 3:** Oviposition and mortality rates of *P. argentipes* and *S. schwetzi* females fed experimentally

	Histamine				Serotonin			
	*P. argentipes*		*S. schwetzi*		*P. argentipes*		*S. schwetzi*	
	Exp	Con	Exp	Con	Exp	Con	Exp	Con
Perctent ovipositing females^a^	90 (79/88)	89 (82/92)	92 (49/53)	96 (51/53)	96 (97/101)	96 (100/104)	85 (40/47)	88 (38/43)
Median no. of eggs per female (range) [IQR]^b^	32 (1–58) [23–40]	31 (1–68) [15–41]	62 (1–128) [47–87]	84 (1–143) [68–101]	32 (1–62) [26–41]	43 (1–74) [33–49]	58 (1–108) [36–70]	70 (2–105) [50–84]
Percent mortality	26 (23/88)	18 (17/92)	72 (38/53)	62 (33/53)	36 (36/101)	27 (28/104)	49 (23/47)	49 (21/43)

^a^Calculated from total sum of blood fed females

^b^The difference between 25th and 75th percentile

*Abbreviations*: Exp, experimental group; Con, control group; IQR, interquartile range

No significant differences were detected in mortality rates between the experimental and the control groups (Table 3).

### Feeding on repeatedly exposed hamsters: effects on sand fly oviposition and mortality

In total, 312 and 318 *P. argentipes* females fed on immunized or naive hamsters, respectively, were separated into glass vials for monitoring of oviposition and mortality. The prevalence of ovipositing females was 58% (181/312) for sand flies fed on immunized hamsters and 60% (192/318) for those fed on naive hamsters (Table 4). There was no significant difference between the numbers of eggs laid by females fed on immunized or naive hamsters. The median number of eggs laid per sand fly female was the same for both groups, 35, with a minimum of 1 egg per female for both groups and maxima of 72 and 74 eggs for the groups fed on immunized and naive hamsters, respectively (Table 4). Immunized hamsters showed very high levels of IgG antibodies against *P. argentipes* salivary proteins: the mean ODs for immunized and naive groups were 2.01 (95% CI: 1.78–2.25) and 0.04 (95% CI: 0.02–0.05), respectively.

**Table 4:** Oviposition and mortality rates of *P. argentipes* females fed on host

	Immunized hamsters	Naive hamsters
Percent ovipositing females^a^	58 (181/312)	60 (192/318)
Median no. of eggs per female (range) [IQR]^b^	35 (1–72) [16–48]	35 (1–74) [9–50]
Percent mortality	63 (197/312)	70 (222/318)

^a^Calculated from total sum of blood fed females

^b^The difference between 25th and 75th percentile

At the end of the experiment (on day 8 post-blood meal) the mortality of sand fly females fed on immunized and naive hamsters did not differ (*χ*^2^ = 2.85, *df* = 1, *P* = 0.09), with mortality rates of 63% (197/312) and 70% (222/318), respectively (Table 4).

## Discussion

Sand flies inoculate salivary molecules including yellow-related proteins into their host’s skin in order to counteract the host haemostatic system and bind biogenic amines such as serotonin and histamine [6, 7]. *Phlebotomus argentipes* has only a single yellow-related protein, PagSP04 [27], and here we demonstrated that it acts as a poor binder of histamine but as a strong binder of serotonin. Similarly, strong affinities for serotonin and weak affinities for histamine have been shown for yellow related proteins of *P. pernicious*, *P. orientalis* and *L. longipalpis* [6, 7]. Our findings support the hypothesis that by binding serotonin, yellow-related proteins take part in counteracting the mammalian haemostatic system, especially platelet aggregation and vasoconstriction. On the other hand, the role of histamine at the site of bite is questionable, and it seems that in mosquito-induced itching in mice, histamine did not play a primary role [28]. In mosquitoes, D7 salivary proteins were shown to bind histamine in addition to other amines [29], but this has not yet been demonstrated for D7-related proteins in sand flies [30]. In sand flies, the D7 proteins are functionally and structurally similar to mosquito D7 proteins. However, the C-terminal domain of sand fly D7 protein is missing major elements of the putative ligand-binding pocket and therefore is not able to bind small molecule ligands [30].

The membrane feeding of *P. argentipes* on blood mixed with serotonin resulted in reduced fecundity (26% fewer eggs than the control group), which suggests that extremely elevated concentrations of serotonin negatively affect *P. argentipes* oviposition. In repeatedly-bitten hosts, however, serotonin concentrations are probably lower than our experimental concentration, and we expect that sand flies are able to cope with these lower concentrations. This corresponds with the results of experimental feeding on hamsters immunized by repeated sand fly bites: *P. argentipes* females did not show any difference in mortality and numbers of laid eggs when experimental and control groups were compared, despite the high levels of anti-saliva antibodies in repeatedly-bitten hamsters.

So far, studies focused on the effects of anti-saliva antibodies on various biological aspects of sand flies have failed to yield consistent results. Ghosh et al. [11] reported that feeding of *P. argentipes* on immunized hamsters led to a gradual decrease of feeding attraction, while mortality increased during subsequent bites. Although hamsters were exposed to sand flies using a similar immunization scheme as in our experiment (to about 90–150 females twice a week followed by a two-week interval, for a total of six exposures), antibody titres detected by those authors were not high. This was explained by low concentrations of each protein fraction in whole saliva, so antibodies developed against these proteins could not have reached high levels [11]. However, in laboratory and in field conditions it was already proved that animals repeatedly exposed to sand flies revealed increased levels of anti-saliva IgGs when compared to the control group [1]. In our experiments, hamsters were exposed to about 100–290 *P. argentipes* females six times at 7–15-day intervals, and antibody titres were very high compared to the control group. In *L. longipalpis*, Vilela et al. [12] reported that females fed on animals immunized by repeated bites obtained lesser amounts of haemoglobin, laid fewer eggs and had higher mortality than females fed on naive animals [12]. On the contrary, Tripet et al. [14] showed that egg production by *L. longipalpis* is not affected by feeding on immunized hosts, and studies on *P. duboscqi* and *P. perniciosus* also did not observe any differences in oviposition or mortality between experimental and control groups of sand flies [13, 15]. Moreover, it is known that sand fly colonies thrive even on laboratory hosts that have been repeatedly exposed to sand flies [20]. Taken together, the effects of anti-saliva antibodies on sand fly physiology are not clear. A more promising approach to altering vector fecundity and mortality might be the immunization of hosts with body tissues, such as whole gut extracts or midgut chitinase [31, 32].

We successfully expressed and purified two yellow-related proteins in *S. schwetzi*, but the ligand binding analysis did not show any affinity to serotonin or histamine. As the feeding preferences of *S. schwetzi* are distinctly different than in *P. argentipes* and other sand fly species studied previously [6, 7], the different properties of this reptile-biting species are not surprising. Adaptations to feeding on either warm-blooded vertebrates or cold-blooded vertebrates [19] may result in different properties for salivary proteins, as demonstrated recently for the relatively low enzymatic activities of apyrase and hyaluronidase in *S. schwetzi* saliva [33]. Unlike in *P. argentipes*, the non-physiologically high concentration of serotonin did not have any effect on *S. schwetzi* fitness. The degradation of serotonin is connected with oxidative stress [34], similarly to heme detoxification [35]. The midgut epithelium of blood-sucking insects is protected from these toxins by the peritrophic matrix (PM) [35], which differs between *S. schwetzi* and *P. argentipes* in morphology and duration: in *S. schwetzi* the PM is thicker and has a prolonged persistence [36], and thus could block the unfavourable effects of serotonin on oviposition.

Surprisingly little is known about the presence of biogenic amines in reptiles. So far, circulating serotonin has been described in three reptilian species, two of them with true or partial endothermy (the leatherback sea turtle, *Dermochelys coriacea*, and the American alligator, *Alligator mississippiensis*). These findings support the hypothesis that circulating serotonin might have emerged with endothermic vertebrates [37]. A study carried out on the common snapping turtle (*Chelydra serpentina*), showed that the release of histamine from basophils takes 40–60 minutes, regardless of antigen concentrations [38]. In contrast, the histamine release from human basophiles is usually completed within several minutes [39, 40]. Due to the fact that sand flies can finish a blood meal within several minutes [14, 41], it is possible that the neutralization of histamine in cold-blooded animals is not necessary.

## Conclusions

We confirmed the high affinity of salivary yellow-related proteins to serotonin in *P. argentipes*, a vector known for its mammalian host preference. This interaction may play a role in the neutralisation of serotonin at the site of the bite and thus facilitate successful blood-feeding. The production of high levels of specific antibodies in hosts repeatedly exposed to *P. argentipes* did not lead to a deterioration of sand fly fitness, suggesting a minor effect of anti-saliva antibodies on sand fly feeding processes. No affinity of the yellow-related proteins to biogenic amines was demonstrated in the reptile biter *S. schwetzi*, and this may reflect the adaptation to cold blooded vertebrates. However, further studies are needed to unravel the role of *Sergentomyia* yellow-related proteins.

## Data Availability

Data supporting the conclusions of this article are included within the article. The datasets used and analyzed during the present study are available from the corresponding author upon reasonable request.

## Abbreviations

PagSP04: *P. argentipes* yellow-related recombinant protein
RNA: ribonucleic acid
DNA: deoxyribonucleic acid
PCR: polymerase chain reaction
YRP: yellow-related protein
PBS: phosphate-buffer saline
MW: molecular weight
MST: microscale thermophoresis
ELISA: enzyme-linked immunosorbent assay
SGH: salivary gland homogenate
OD: optical density
Kd: dissociation constant
CI: confidence interval
SschwYRP: *S. schwetzi* yellow-related recombinant protein
